# Dynamic Cobalt Leaching‐Redeposition Equilibrium in Co_9_S_8_/Co‐Ni_3_S_2_ Electrocatalysts Boosts Oxygen Evolution Activity

**DOI:** 10.1002/advs.77217

**Published:** 2026-08-18

**Authors:** Xue‐Min Chen, Meng‐Ya Zhao, Xiu‐Li Zhao, Hui‐Ying Mu, Yue Zhou, Dong‐Ya Ma, Fa‐Tang Li

**Affiliations:** ^1^ Hebei Provincial Key Laboratory of Photoelectric Control on Surface and Interface College of Science Hebei University of Science and Technology Shijiazhuang China; ^2^ College of Energy Storage Technology Shandong University of Science and Technology Qingdao China

**Keywords:** electrocatalyst, leaching‐redeposition, oxygen evolution reaction, transition metal composite

## Abstract

Developing catalysts that provide high catalytic activity and durability is critical for practical water electrolysis. However, most current studies focus on activity origins, overlooking the role of metal ion leaching and redeposition in catalytic stability under long‐term operation. Herein, we synthesized cobalt‐nickel sulfide composites (Co_9_S_8_/Co‐Ni_3_S_2_) and investigated the dynamic changes in their reaction sites, particularly the leaching‐redeposition processes in the oxygen evolution reaction (OER). Characterization analysis indicates that Co and Ni ions leach from the surface of Co_9_S_8_/Co‐Ni_3_S_2_, combine with OH^−^ in the electrolyte, and subsequently redeposit onto the catalyst surface, where they are further electrooxidized to CoOOH and NiOOH, thus boosting performance. Specifically, the leaching‐redeposition of Co exerts a dominant effect on boosting catalytic activity and stability. Additionally, dynamic equilibrium roughens the surface, improving electroactive area and mechanical stability. Density functional theory (DFT) calculations confirm a lattice oxygen‐mediated mechanism, and the composite exhibits an optimized Co d‐band center with strong Ni‐Co 3d orbital overlap, enabling optimal intermediate adsorption and electron transfer. When assembled into an anion exchange membrane water electrolyzer (AEMWE), Co_9_S_8_/Co‐Ni_3_S_2_ operates for 500 h at 100 mA cm^−2^ under an applied potential of 1.59 V, outperforming most reported electrocatalysts. These findings offer design insights for durable OER electrocatalysts.

## Graphical abstract

Reconstruction of a Co_9_S_8_/Co‐Ni_3_S_2_ electrocatalyst stabilizes via a leaching‐redeposition dynamic equilibrium of metal ions. Interestingly, the Co‐ion leaching and redeposition play a crucial role in boosting oxygen evolution performance. This endogenous self‐repair cycle continuously offers active sites, allowing the catalyst to maintain superior OER kinetics even under prolonged operation.

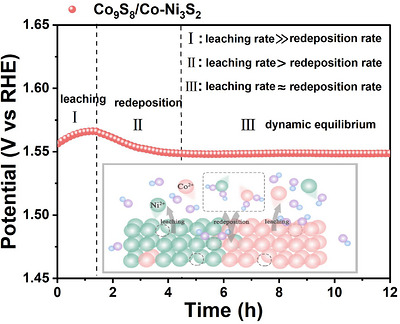

## 1 Introduction

Electrocatalytic water splitting stands as a promising strategy for the large‑scale generation of sustainable hydrogen. However, the inferior reaction dynamics of the anodic OER remain a critical bottleneck limiting overall efficiency [1, 2, 3]. While various OER catalysts, including transition metal hydroxides, oxides, sulfides, etc., have been exploited to boost reaction kinetics, achieving prolonged stability under practical industrial conditions still poses a formidable challenge [4, 5]. It is highly desirable to create OER catalysts combining remarkable activity and durability. It is crucial to understand the fundamentals of the dynamic evolution of metal active sites during prolonged catalytic operation.

Extensive research has demonstrated that the surface of transition metal‐based (Ni, Co, Fe) electrocatalysts undergoes electrochemical reconstruction during OER, forming metal oxyhydroxides (MOOH), known to be the actual active phases [6, 7, 8]. However, the influence of surface metal ion leaching and redeposition on catalytic activity and stability is often underestimated. Zhang et al. found that for the alkaline hydrogen evolution reaction (HER) electrocatalyst Ni_4_Mo, Mo is unstable and leaches as MoO_4_^2−^, which then re‐adsorbs and polymerizes. Adding MoO_4_^2−^ to the electrolyte restores Ni_4_Mo durability and enhances HER activity of Ni, Fe, and Co [9]. Previous studies have demonstrated that NiFe‐based OER electrocatalysts can achieve self‐repair through dynamic Fe ion exchange between the surface of the catalyst and electrolyte [10]. Li et al. further reported that when the rate of Fe^n+^ deposition onto a host material exceeds dissolution, highly efficient and robust dynamic electrochemical interfaces can be constructed for long‐term operation [11]. Recently, Boettcher and co‐workers also demonstrated that surface‐confined Fe cations on Ni and Co (oxy)hydroxides, introduced via a controlled chronoamperometric spiking method, display exceptional OER activity through a cooperative Fe─O─Fe adsorbate‐evolving mechanism (AEM) pathway [12]. While these pioneering studies have firmly established the significance of trace Fe exchange between the electrolyte and host matrix, the potential of key transition metals other than Fe (such as Co) to undergo an endogenous and self‐sustained dynamic leaching‑redeposition equilibrium in structural precatalysts remains unexplored. Unlocking this intrinsic, self‐sustained dynamic activity and understanding its contribution to durability are pivotal for designing next‐generation OER electrocatalysts.

Herein, the Co_9_S_8_/Co‐Ni_3_S_2_ composite electrocatalyst was synthesized via a one‐step method. Its superior overall performance was further corroborated by comparison with other advanced nickel‐based electrocatalysts (Table S1), and the origins of its remarkable catalytic activity and stability were systematically explored. Comprehensive characterization reveals that prolonged cycling induces surface roughening, which enhances mechanical stability. More importantly, we identify a dynamic leaching‐redeposition equilibrium involving Co and Ni ions as the key mechanism governing catalytic performance: Co and Ni ions leach into the electrolyte, combine with OH^−^ ions, redeposit onto the catalyst surface, and then are oxidized to CoOOH and NiOOH, thereby boosting catalytic performance. Specifically, the leaching‐redeposition of Co exerts a dominant effect on boosting catalytic activity and stability. DFT calculations illustrate that the optimized d‐band center and strong orbital hybridization in Co_9_S_8_/Co‐Ni_3_S_2_ improve intermediate adsorption and facilitate electron transfer. Moreover, the catalyst favors the lattice oxygen‐mediated mechanism (LOM) with a much lower rate‐determining energy barrier than that of the AEM pathway, which accounts for its outstanding OER activity. This work demonstrates the existence of a dynamic leaching‐redeposition equilibrium, with Co ions playing a dominant role, offering new insights for constructing efficient and durable OER electrocatalysts.

## 2 Results and Discussion

### 2.1 Dynamic Equilibrium of Metal Ions Leaching and Redeposition

**FIGURE 1:**
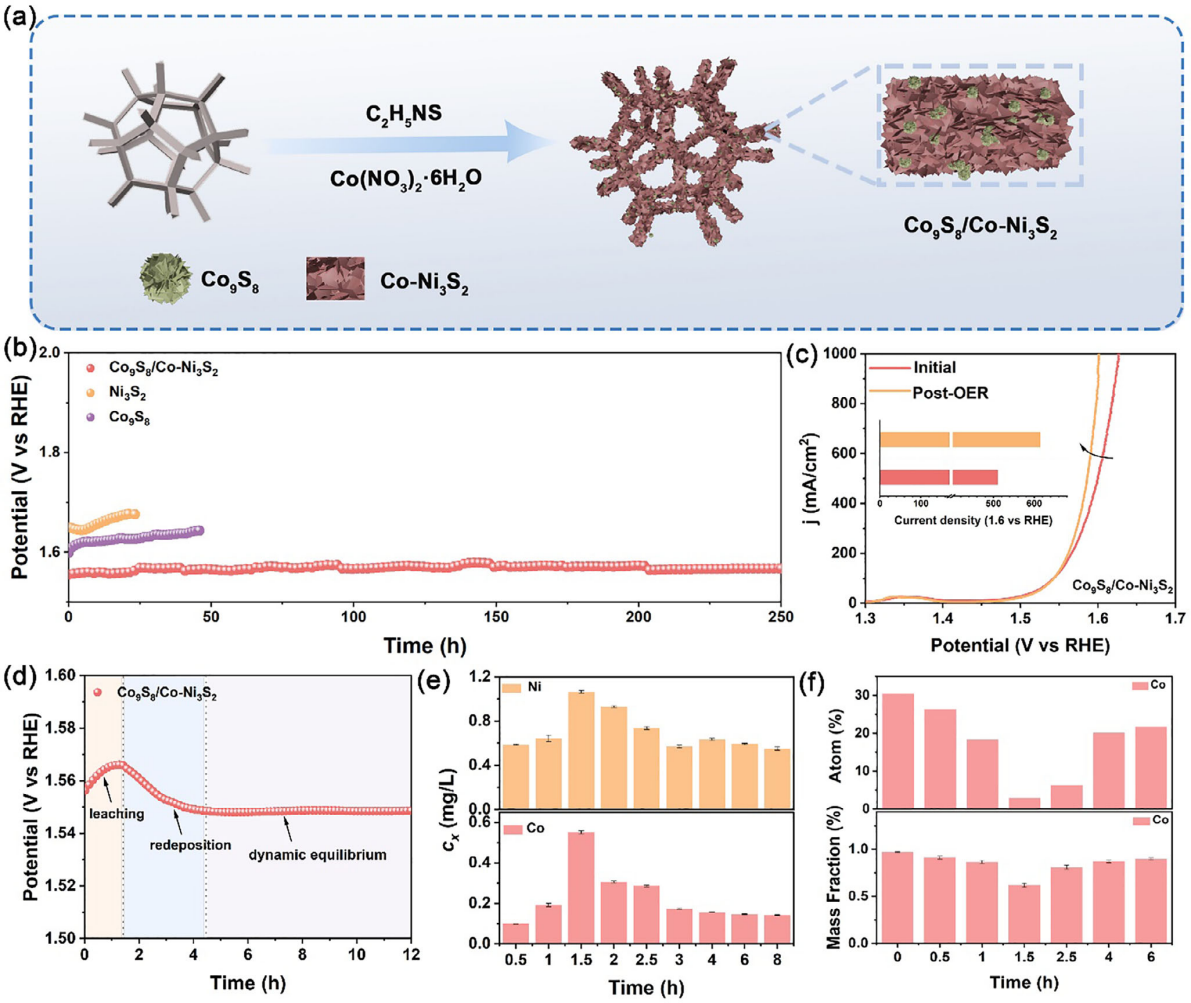
(a) Schematic diagram of the preparation of Co_9_S_8_/Co‐Ni_3_S_2_ electrode material. (b) Chronopotentiometry curves of Ni_3_S_2_, Co_9_S_8_, Co_9_S_8_/Co‐Ni_3_S_2_ at 100 mA cm^−2^. (c) Initial and post‐OER LSV plots of Co_9_S_8_/Co‐Ni_3_S_2_. (d) Chronopotentiometry curves of the Co_9_S_8_/Co‐Ni_3_S_2_ catalyst at 50 mA cm^−2^ (without prior CV activation). (e) Time‐dependent concentration of Co and Ni ions leached from Co_9_S_8_/Co‐Ni_3_S_2_ into KOH electrolyte. (f) Time‐dependent semi‐quantitative EDS (top) and quantitative ICP‐MS (bottom) analyses of Co content in the Co_9_S_8_/Co‐Ni_3_S_2_ catalyst.

The nickel foam‐supported Co_9_S_8_/Co‐Ni_3_S_2_ electrocatalyst was synthesized via a hydrothermal method, using nickel foam and cobalt nitrate as the nickel and cobalt sources, respectively. A schematic illustration of the synthesis process is shown in Figure 1a. The Co_9_S_8_/Co‐Ni_3_S_2_ composite features a structure with Co‐Ni_3_S_2_ nanosheets at the bottom and Co_9_S_8_ nanosheet‐derived nanocorals on the top (Figures S1–S3, S6 and S7). HAADF‐STEM image and associated element mapping images illustrate the spatial dispersion of Ni, S, and Co across the composite material (Figure S5) [13, 14]. Phase analysis via the XRD patterns shown in Figure S8 validates the coexistence of Co_9_S_8_, Ni_3_S_2_, and the metallic Ni foam support. Relative to pristine Ni_3_S_2_, the slight deviation of the (110) peak to smaller diffraction angles in the Co_9_S_8_/Co‐Ni_3_S_2_ composite stems from the incorporation of Co atoms into Ni sites during crystal growth [15, 16]. This result was further confirmed by HRTEM observations [17, 18] (Figure S4). X‐ray photoelectron spectroscopy (XPS) analysis reveals that, compared with pristine Ni_3_S_2_, the Ni 2p peaks in the Co_9_S_8_/Co‐Ni_3_S_2_ composite shift toward higher binding energies (Figure S9). This indicates the incorporation of Co atoms into the Ni_3_S_2_ lattice, which alters the local electronic structure of Ni and elevates it to a higher oxidation state [19].

The electrocatalytic OER activity of the prepared catalysts was assessed in a 1.0 m KOH electrolyte using a standard three‐electrode system (Figure S10). Notably, the Co_9_S_8_/Co‐Ni_3_S_2_ catalyst exhibited a low overpotential of 280 mV at 50 mA·cm^−2^ and a Tafel slope of 76.4 mV·dec^−1^, outperforming most reported peer catalysts (Figure S11 and Table S2) and highlighting its potential as a highly active anode for water splitting. To evaluate its potential for practical applications, the electrochemical durability was tested at 100 mA·cm^−2^ (Figure 1b). The Co_9_S_8_/Co‐Ni_3_S_2_ electrode showed exceptional stability, maintaining steady operation for over 250 h, which compares favorably with recently reported Ni‐based catalysts (Table S3). Interestingly, post‐stability LSV curves revealed an obvious enhancement in OER activity for Co_9_S_8_/Co‐Ni_3_S_2_ compared to its fresh state (Figure 1c). Conversely, pristine Ni_3_S_2_ and Co_9_S_8_ underwent severe activity attenuation under identical conditions (Figures S12 and S13). To gain insights into this activation behavior, the evolution of the catalyst was monitored directly without prior CV footprint activation (Figure 1d). The chronopotentiometric potential response at 50 mA·cm^−2^ exhibited a distinct three‐stage profile over a 12‐h test: an initial increase, a subsequent decline, and final stabilization. Guided by previous findings [20], this behavior is attributed to an in situ surface reconstruction process, where metal ions initially leach into the electrolyte and then redeposit onto the electrode, eventually reaching a dynamic dissolution‐deposition equilibrium at the reaction interface.

To monitor the in situ surface reconstruction, the concentrations of leached metal ions in the electrolyte at various OER stages were measured by ICP‐MS. As shown in Figure 1e, the nickel and cobalt ion concentrations gradually increased during the initial 1.5 h of the OER, followed by a gradual decrease until reaching a plateau at 3 h. This trend indicates that metal ions initially leach from the Co_9_S_8_/Co‐Ni_3_S_2_ surface and subsequently redeposit, establishing a dynamic leaching‐redeposition equilibrium. Morphologically, the initial composite of nanosheets and nanocorals gradually merged into a rough granular surface after 3 h of OER testing (Figure S14), a transition that improves the mechanical robustness of the catalyst. As revealed by the elemental mapping (Figure S15), the signal intensity of Co gradually weakened during the initial 1.5 h of the OER, but subsequently strengthened as the reaction proceeded. This evidence suggests that Co ions undergo a process of initial dissolution followed by redeposition during the OER. Additionally, semi‐quantitative Energy Dispersive Spectrometer (EDS) characterization (the top panel of Figure 1f) showed that the atomic percentage of Co on the Co_9_S_8_/Co‐Ni_3_S_2_ catalyst surface initially decreased and subsequently increased with the progress of the OER, showing a trend opposite to the Co ion concentration in the electrolyte. Bulk ICP‐MS analysis of digested catalysts (Figure 1f) further revealed that the Co mass fraction initially decreased and then increased with OER duration. This V‐shaped trend once again confirms the dynamic leaching‐redeposition mechanism of active metal sites during OER.

**FIGURE 2:**
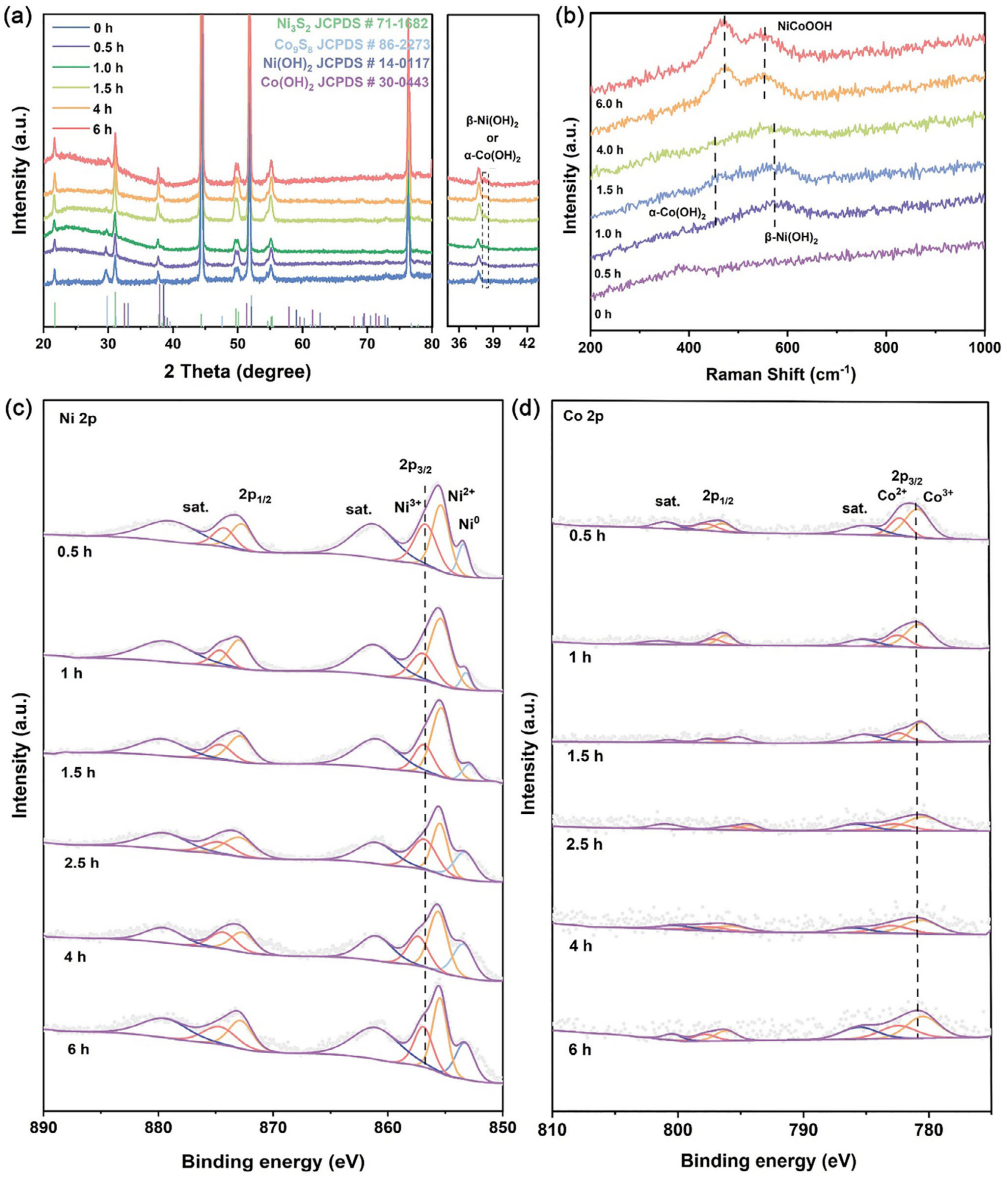
Characterization of the Co_9_S_8_/Co‐Ni_3_S_2_ catalyst after various OER durations: (a) XRD patterns, (b) Raman spectra, and (c‐d) XPS spectra.

Subsequently, ex situ XRD, Raman, and XPS characterizations were performed on the Co_9_S_8_/Co‐Ni_3_S_2_ catalysts harvested after different OER durations to monitor the phase evolution, reconstruction pathways, and active species. Notably, with increasing OER duration, diffraction peaks corresponding to α‐Co(OH)_2_ and β‐Ni(OH)_2_ gradually emerged (Figure 2a). It is speculated that during the OER process, the leached Ni and Co ions react with the KOH electrolyte to form their corresponding hydroxides, which subsequently redeposit onto the catalyst surface. Consistent structural variations were also observed in the Raman spectra (Figure 2b). The pristine Co_9_S_8_/Co‐Ni_3_S_2_ catalyst showed no prominent Raman signals. However, a broad peak at 580 cm^−1^ became apparent after 0.5 h of continuous OER, attributable to the vibration of β‐Ni(OH)_2_ [21]. This suggests that leached Ni ions react with the KOH electrolyte to form nickel hydroxide, which subsequently deposits onto the catalyst surface. Prolonging the OER duration to 1 h resulted in an additional peak at 455 cm^−1^ associated with the A_1g_ vibration of α‐Co(OH)_2_ [22], verifying a similar leaching‐redeposition process for Co ions. As the reaction proceeded to 4 h, two distinct peaks at 470 and 550 cm^−1^ became prominent, signaling the phase transition into active NiOOH and CoOOH species. Semi‐quantitative analysis of the deconvoluted Co 2p and Ni 2p XPS spectra reveals a continuous decline in the Ni^2+^/Ni^3+^ and Co^2+^/Co^3+^ ratios upon prolonged OER testing (Figure 2c,d and Table S1), signifying the gradual increase of high‐valence active species. Based on these experimental findings and previous literature [23], the Co_9_S_8_/Co‐Ni_3_S_2_ catalyst is demonstrated to undergo a dynamic reconstruction pathway during OER: *M^z+^* (*leached*) + *z*OH^−^ ⇌ *M*(OH)*_z_* (*redeposited*) $\overset{electrooxidation}{\to }$ *M*OOH (*active species*). Crucially, this unique dynamic equilibrium of active metal ion leaching and redeposition plays a pivotal role in dramatically boosting the electrochemical performance of the Co_9_S_8_/Co‐Ni_3_S_2_ catalyst.

**FIGURE 3:**
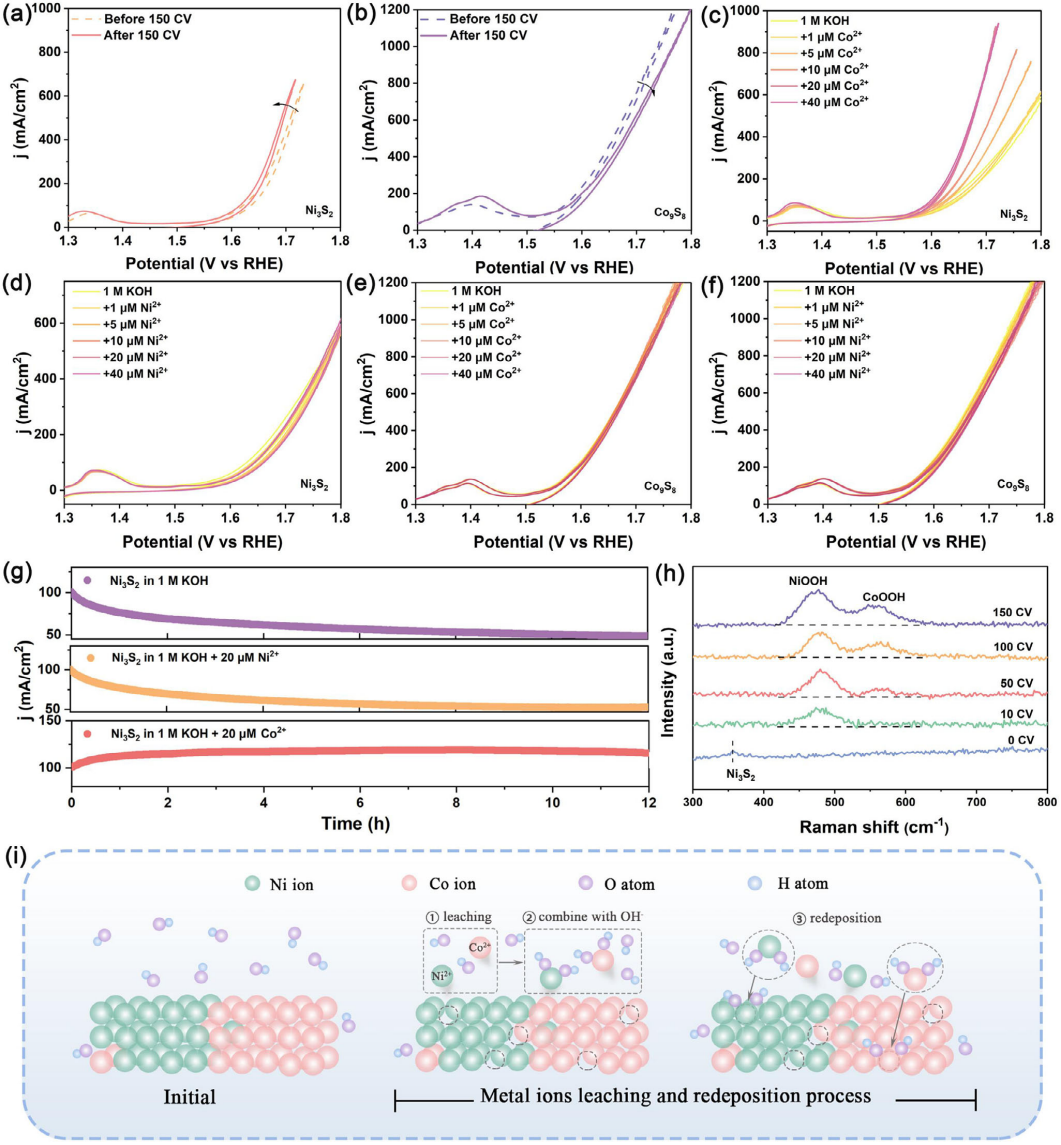
(a‐b) CV curves of Ni_3_S_2_ and Co_9_S_8_ before and after 150 cycles in KOH after testing Co_9_S_8_/Co‐Ni_3_S_2_. (c‐d) CV curves of Ni_3_S_2_ in 1 m KOH electrolyte with different concentrations of Co^2+^ and Ni^2+^. (e‐f) CV curves of Co_9_S_8_ in 1 m KOH electrolyte with different concentrations of Co^2+^ and Ni^2+^. (g) Current density‐time curves under different conditions. (h) Raman spectra of Ni_3_S_2_ electrodes in KOH electrolyte with 20 µm Co^2+^ after different numbers of CV cycles (0 to 150 cycles). (i) Schematic illustration of metal ion leaching and redeposition process.

To verify the contribution of leached nickel and cobalt ions to the OER process, a series of comparative experiments were performed. CV curves of pristine Ni_3_S_2_ and Co_9_S_8_ pre‐ and post‐ 150 CV cycles in metal ion‐free KOH and in KOH after testing Co_9_S_8_/Co‐Ni_3_S_2_ were separately measured (Figure 3a,b and Figure S16). Obviously, in the metal ions‐free KOH electrolyte, the activity of Ni_3_S_2_ and Co_9_S_8_ both decreased after 150 CV cycles. However, in the KOH after testing Co_9_S_8_/Co‐Ni_3_S_2_, Ni_3_S_2_ showed enhanced activity after 150 CV cycles, whereas Co_9_S_8_ did not improve. This indicates that the leached metal ions in the KOH electrolyte can deposit onto the Ni_3_S_2_ surface and enhance its catalytic activity, but the dissolved metal ions do not respond to Co_9_S_8_. Subsequently, CV tests were performed on Ni_3_S_2_ and Co_9_S_8_ in KOH electrolytes containing different concentrations of Co^2+^ and Ni^2+^ ions (Figure 3c–f). The results indicate that increasing the Ni^2+^ concentration had little effect on the electrocatalytic activity of either material. In contrast, elevating the Co^2+^ concentration significantly enhanced the activity of Ni_3_S_2_, while exerting no noticeable influence on that of Co_9_S_8_. Based on the above results, it can be inferred that the leaching‐redeposition process of Co ions plays a crucial role in enhancing the OER activity.

Furthermore, the long‐term stability of Ni_3_S_2_ was investigated in different KOH electrolytes. The results (Figure 3g) show that the current density decayed in pure KOH, with a similar trend in KOH containing 20 µm Ni^2+^. In contrast, in the KOH electrolyte containing Co^2+^, the current density of Ni_3_S_2_ did not decrease but instead increased gradually and finally reached a steady state. Thus, the dynamic Co^2+^ leaching and redeposition equilibrium established at the catalyst surface is shown to promote Ni_3_S_2_ catalytic activity and operational stability. To validate the proposed mechanism whereby dissolved Co^2+^ ions gradually deposit on Ni_3_S_2_ during the OER and form the highly active CoOOH and NiOOH species, this evolution was investigated through the application of quasi‐in situ Raman spectroscopy (Figure 3h). The spectra revealed that after 50 CV cycles, characteristic Raman peaks corresponding to both CoOOH and NiOOH emerged on the Ni_3_S_2_ surface, thus providing clear evidence for the deposition of electrolyte‐added Co ions onto the Ni_3_S_2_ surface. In light of these findings, it is inferred that during the OER process, Co and Ni ions from the Co_9_S_8_/Co‐Ni_3_S_2_ surface leach into the electrolyte, react with OH^−^, and subsequently redeposit onto the catalyst, establishing a dynamic leaching‐redeposition equilibrium (Figure 2i). It is worth noting that the dynamic process in the Co_9_S_8_/Co‐Ni_3_S_2_ system is entirely endogenous and self‐sustained. The self‐leached Co ions from the precatalysts spontaneously assembly a robust and highly active reconstructed interface without needing external metal precursors. This endogenous self‐repair cycle continuously refreshes the active sites, allowing the catalyst to maintain superior OER kinetics even under prolonged operation.

**FIGURE 4:**
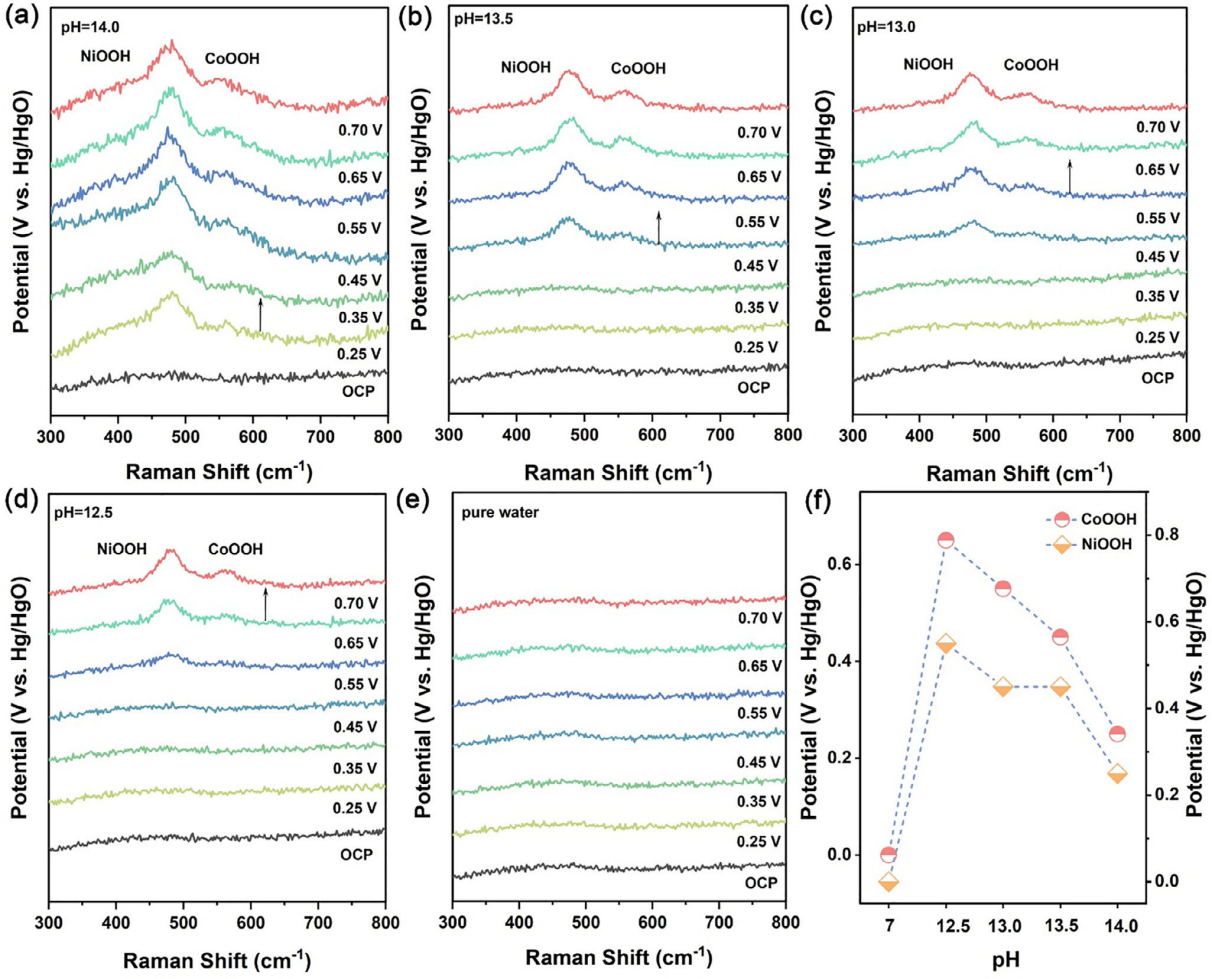
Potential‐dependent in situ Raman spectra of the Co_9_S_8_/Co‐Ni_3_S_2_ catalyst in KOH electrolyte with different pH values: (a) 14.0, (b) 13.5, (c) 13.0, and (d) 12.5. (e) Formation potentials of CoOOH and NiOOH species as a function of electrolyte pH.

As the leaching‐redeposition process of metal ions in the Co_9_S_8_/Co‐Ni_3_S_2_ catalyst requires the participation of hydroxide ions, we speculate that the OER process of this catalyst is highly dependent on pH. Moreover, previous literature has established that local pH variations at the electrode‐electrolyte interface act as one of the key factors for CoO_x_ catalyst surface reconstruction [24]. Consequently, in situ Raman spectra were utilized to track the structural evolution of the Co_9_S_8_/Co‐Ni_3_S_2_ catalyst across electrolytes with varying pH levels. As shown in Figure 4, in a strongly alkaline electrolyte (pH = 14 KOH), the formation of active CoOOH and NiOOH species is initiated at a low potential of only 0.25 V. However, with the continuous decrease in electrolyte pH, the formation potentials of the active CoOOH and NiOOH species shift upward. At pH values of 13.5, 13.0, and 12.5, the formation potentials are 0.45, 0.55, and 0.65 V for CoOOH species, and 0.45, 0.45, and 0.55 V for NiOOH, respectively. Strikingly, when evaluated in near‐neutral deionized water, the Co_9_S_8_/Co‐Ni_3_S_2_ catalyst fails to yield any active species during the OER. These findings prove that the leaching‐redeposition equilibrium of metal ions in the Co_9_S_8_/Co‐Ni_3_S_2_ catalyst during the OER is highly pH‐dependent. A higher electrolyte pH dynamically accelerates this equilibrium process, ultimately boosting both catalytic activity and long‐term stability.

To explore the generality of the dynamic leaching‐redeposition equilibrium of active metal ions, we synthesized NiCo‐LDH/Ni(OH)_2_ and NiO/NiCo_2_O_4_ as representative model catalysts using hydrothermal synthesis and thermal calcination, respectively. As illustrated in Figure S17, both the Ni‐Co‐based hydroxides and oxides exhibit a characteristic chronological potential profile under a constant current density: an initial evolution, a subsequent decay, and ultimately stabilization. This phenomenon is similar to the dynamic leaching‐redeposition of metal ions in Co_9_S_8_/Co‐Ni_3_S_2_ catalyst. Notably, by comparing the CP curves of the three catalysts (Co_9_S_8_/Co‐Ni_3_S_2_, NiCo‐LDH/Ni(OH)_2_ and NiO/NiCo_2_O_4_), it is evident that their timescales for metal ion dissolution and redeposition differ, which is speculated to be closely related to the specific type of NiCo‐based catalyst.

### 2.2 OER Mechanism and DFT Calculations

**FIGURE 5:**
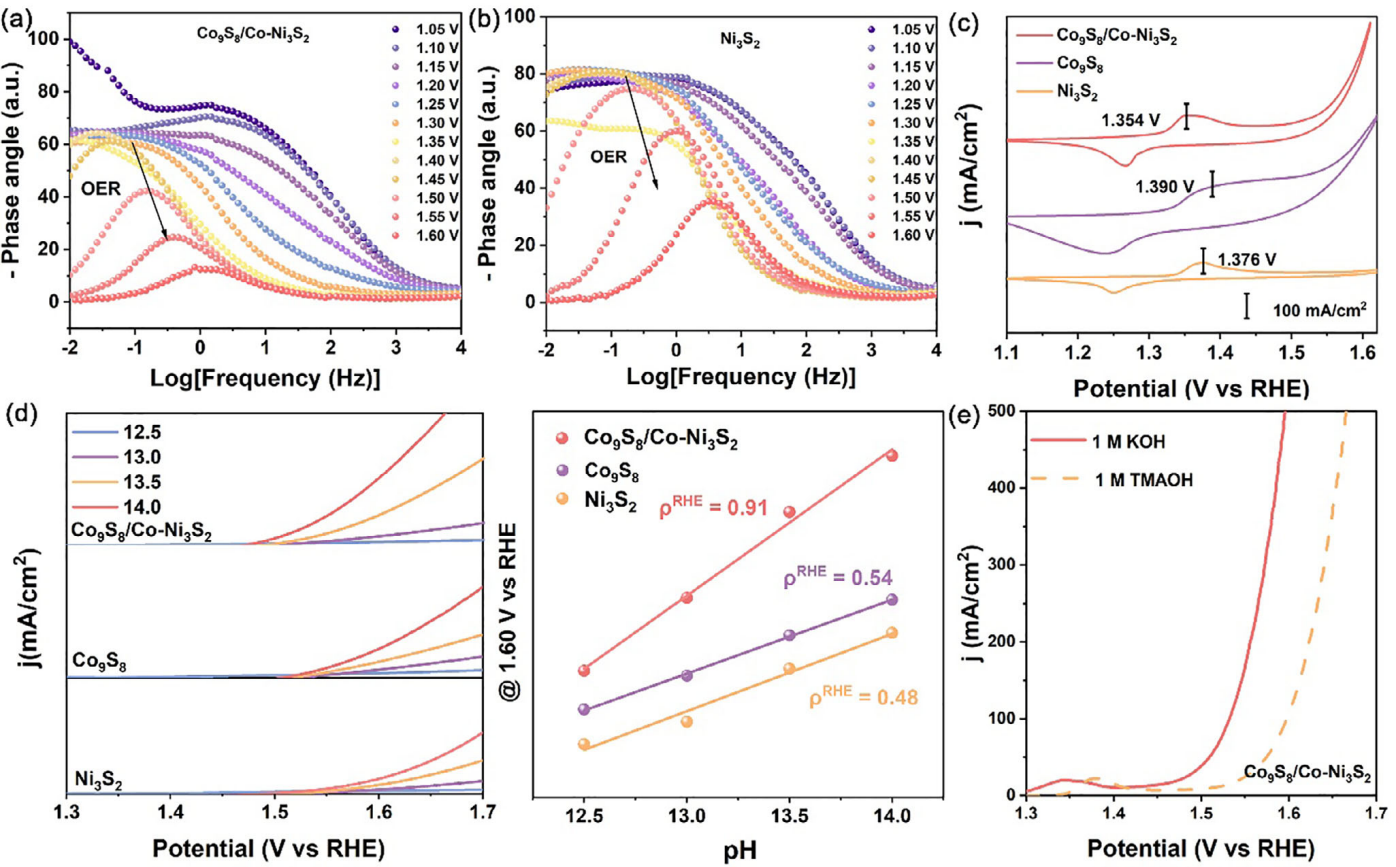
Electrocatalytic mechanism of OER: (a‐b) In situ Bode phase profiles of Co_9_S_8_/Co‐Ni_3_S_2_ and Ni_3_S_2_ for OER. (c) Redox peaks in CV curves of Co_9_S_8_/Co‐Ni_3_S_2_, Co_9_S_8,_ and Ni_3_S_2_. (d) To determine the proton reaction orders (ρ^RHE^ = ∂logi/∂pH), LSV measurements were conducted in KOH media across a range of pH values. The resultant OER current densities (at 1.60 V vs. RHE) were then analyzed via logarithmic correlation with pH. (e) Comparison of LSV plots of Co_9_S_8_/Co‐Ni_3_S_2_ in different alkaline systems.

To elucidate the mechanistic basis for the boosted OER performance of Co_9_S_8_/Co‐Ni_3_S_2_, the double‐layer capacitance (C_dl_) was quantified by performing CV scans at incremental rates within the capacitive potential window. The C_dl_ value for Co_9_S_8_/Co‐Ni_3_S_2_ is 14.14 mF cm^−2^, exceeding those of pristine Ni_3_S_2_ and Co_9_S_8_ (Figures S18–S20). Given that C_dl_ is positively correlated with the electrochemical surface area (ECSA), these findings demonstrate that Co_9_S_8_/Co‐Ni_3_S_2_ possesses a larger ECSA, providing more accessible active sites to accelerate OER kinetics [25]. The Co_9_S_8_/Co‐Ni_3_S_2_ catalyst demonstrates the least resistive barrier for charge transfer among its counterparts, reflecting expedited electron migration and enhanced kinetics at the electrode/electrolyte boundary (Figure S21). In situ Bode analysis, a well‐established technique for probing electrochemical reaction kinetics, substantiates this conclusion (Figure 5a,b and Figure S22). Measurements were conducted over a frequency window of 10^4^ to 10^−2^ Hz with an amplitude of 5 mV. As potential rises, the high‐frequency feature migrates toward the low‐frequency regime, accompanied by the emergence of a low‐frequency peak [26]. This process marks the onset of the electrooxidation reaction. An apparent low‐frequency peak was observed on Co_9_S_8_/Co‐Ni_3_S_2_ at 1.30 V (vs. RHE) for OER, distinctly below the potentials of Ni_3_S_2_ (1.35 V) and Co_9_S_8_ (1.40 V). With progressive potential increase, the low‑frequency peaks move to higher frequencies and show diminished phase angles, an effect attributable to interfacial redox events at the electrode‐electrolyte boundary [27]. Consequently, the Co_9_S_8_/Co‐Ni_3_S_2_ exhibits a lower peak‐formation potential and a sharper decline in phase angle compared with Ni_3_S_2_ and Co_9_S_8_, suggesting more rapid reaction kinetics. The Ni^2+^/Ni^3+^redox peak of Co_9_S_8_/Co‐Ni_3_S_2_ is located at 1.354 V in Figure 5c, shifting to more negative potentials compared with Co_9_S_8_ (1.390 V) and Ni_3_S_2_ (1.376 V), which points to a lower oxidation potential for Ni in the composite [28].

Additionally, the dynamic evolution of active species on the Co_9_S_8_/Co‐Ni_3_S_2_ and Ni_3_S_2_ catalysts during OER was comparatively investigated via in situ Raman spectroscopy (Figure 4a and Figure S23). With the increment of the applied OER potential, two characteristic Raman bands emerge at 457 and 557 cm^−1^ for the Co_9_S_8_/Co‐Ni_3_S_2_ catalyst, which can be attributed to the formation of Ni^3+^‐O and Co^3+^‐O species [29]. This endows the Co_9_S_8_/Co‐Ni_3_S_2_ catalyst with dual active sites (NiOOH and CoOOH), thereby favoring the enhancement of catalytic activity. Notably, the Raman peak for the Ni^3+^‐O species in the Co_9_S_8_/Co‐Ni_3_S_2_ catalyst emerges at (0.25 V vs. Hg/HgO), which is lower than that in pure Ni_3_S_2_ (0.55 V), confirming that nickel oxidation is thermodynamically more favorable within the composite [30]. Furthermore, the underlying mechanisms of Co_9_S_8_/Co‐Ni_3_S_2_, pristine Ni_3_S_2_, and Co_9_S_8_ were determined via pH‑dependent activity measurements. The proton reaction order was determined by plotting the logarithmic OER current density at 1.60 V (vs. RHE) against pH (Figure 5d) [31]. Co_9_S_8_/Co‐Ni_3_S_2_, pristine Ni_3_S_2_, and Co_9_S_8_ exhibited ρ^RHE^ values of 0.91, 0.48, and 0.54, respectively. The significantly higher ρ^RHE^ for Co_9_S_8_/Co‐Ni_3_S_2_ indicates its stronger pH‐dependent OER performance relative to the other two electrocatalysts [32]. The higher pH dependence of Co_9_S_8_/Co‐Ni_3_S_2_ indicates that the composite facilitates the LOM involving a decoupled proton‐electron transfer process [33]. The tetramethylammonium cation (TMA^+^) specifically interacts with the superoxide (O^2−^) intermediate generated via deprotonation and O─O coupling [34]. Accordingly, the identification of O^2−^ species using TMA^+^ verifies the LOM process. As shown in Figure 5e and Figures S24 and S25, Co_9_S_8_/Co‐Ni_3_S_2_ exhibits significantly reduced OER catalytic performance in the TMAOH electrolyte relative to the KOH electrolyte, whereas Ni_3_S_2_ and Co_9_S_8_ exhibit relatively slight changes. These results confirm that the OER on Co_9_S_8_/Co‐Ni_3_S_2_ proceeds via the LOM pathway.

**FIGURE 6:**
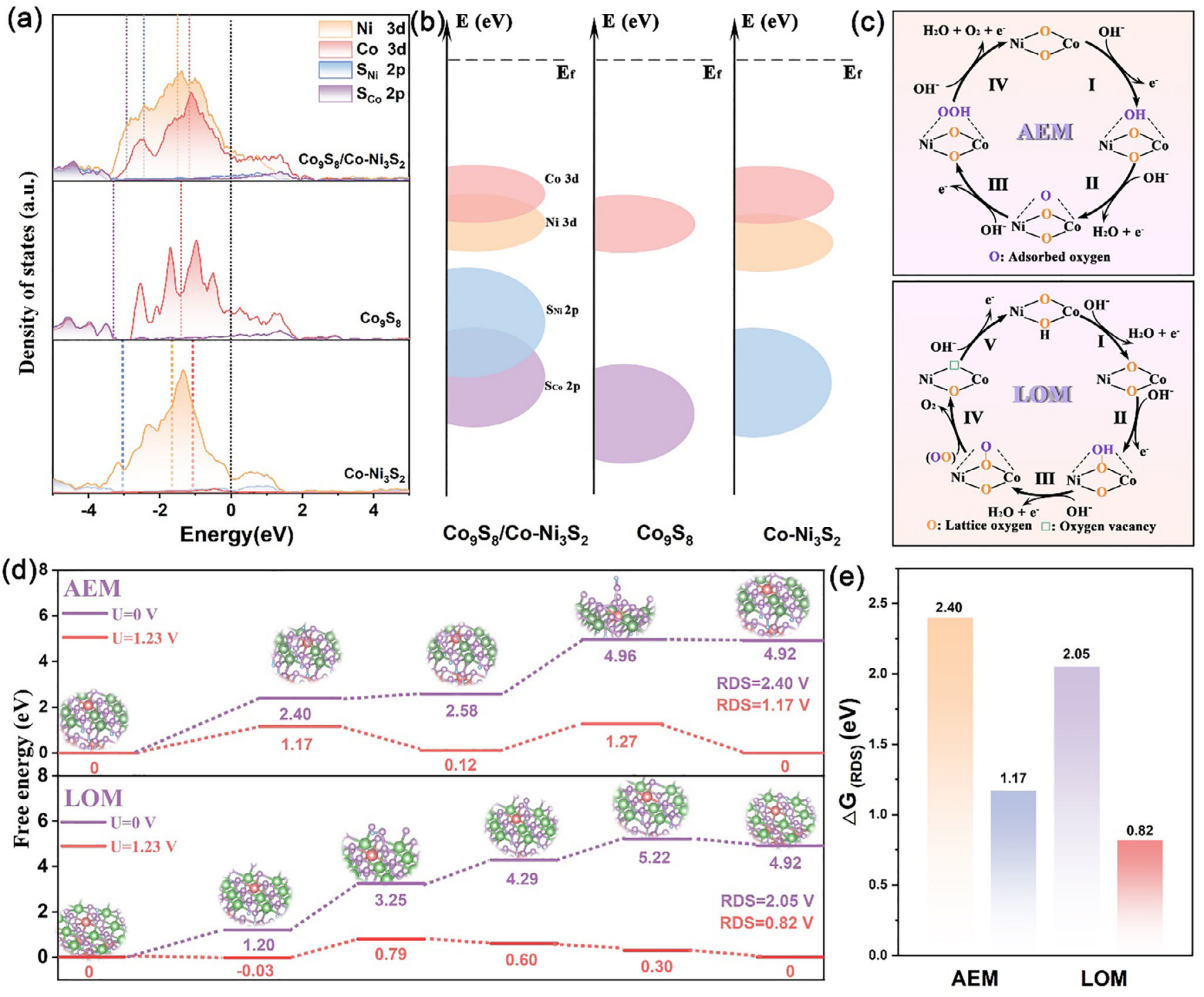
OER mechanism analysis based on DFT calculations: (a) The calculated PDOS and (b) the schematic band diagrams of Co_9_S_8_/Co‐Ni_3_S_2_, Co_9_S_8_ and Co‐Ni_3_S_2_. (c) Proposed reaction routes for the OER governed by AEM and LOM models. (d) Gibbs free energy landscapes for OER via AEM and LOM. (e) Energy barrier histograms for the OER rate‐determining step (RDS) on Co_9_S_8_/Co‐Ni_3_S_2_ via the AEM and LOM mechanisms.

Based on DFT calculations performed on constructed models of the Co_9_S_8_/Co‐Ni_3_S_2_ catalyst, we investigated the underlying OER mechanism (Figure S26). To clarify the underlying reason for the boosted OER activity on Co_9_S_8_/Co‐Ni_3_S_2_, the electronic structure and the role of each constituent element were evaluated by analyzing the partial density of states (PDOS) (Figure 6a,b). The adsorption energies of critical intermediates are primarily modulated by the relative location of the d‐band center. A downshifted d‐band center value indicates weaker adsorption strength, while energies approaching the Fermi level correspond to stronger intermediate binding [35]. Specifically, compared to Ni_3_S_2_ and Co_9_S_8_, the Co d‐band center in the composite is situated at an intermediate position, whereas that for Ni is positioned nearer the Fermi level.

This electronic configuration endows the composite with an optimal binding capability for reaction intermediates. Furthermore, the band diagram reveals substantial overlap between the 3d orbitals of Ni and Co atoms, indicating strong bonding that facilitates electron transfer between them [36]. Simultaneously, the enhanced hybridization of the d‐p band centers in the composite strengthens the covalency of the S─Ni and S─Co bonds, which further promotes electron transfer efficiency and boosts the catalytic activity [37].

Owing to the surface oxidation that occurs during OER, NiOOH and CoOOH were selected as model catalysts to study the OER mechanism and unveil the reaction pathway (Figures S27–S29). The schematic diagram in Figure 6c illustrates the possible AEM and LOM reaction pathways on the catalyst. To examine the advantages of the Co_9_S_8_/Co‐Ni_3_S_2_ composite via different OER pathways in detail, the free‐energy profiles (ΔG) were calculated at applied potentials of 0 and 1.23 V, as shown in Figure 6d. Compared with the AEM pathway, the LOM pathway has a reduced RDS energy barrier of 0.82 eV (^*^O → ^*^OOH) at U = 1.23 V [38], as illustrated by the energy barrier histograms in Figure 6e. Consequently, the LOM pathway endows the electrocatalyst with superior OER activity relative to the AEM pathway.

### 2.3 Overall Water Splitting Performance in an Anion‐Exchange Membrane Electrolyzer

**FIGURE 7:**
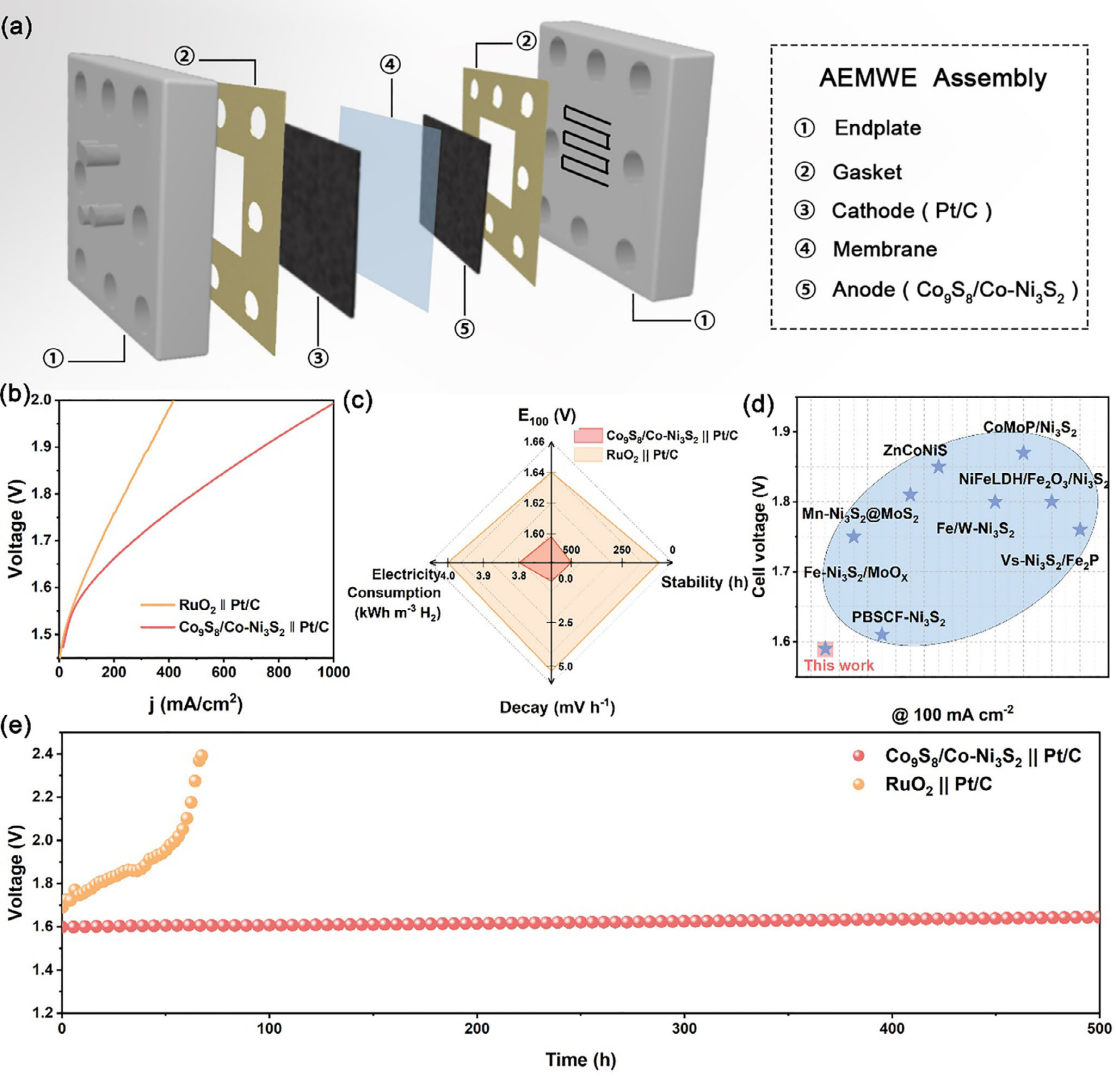
(a) Visual representation of the AEM electrolyzer components and setup. (b) Performance comparison for overall water splitting between the RuO_2_||Pt/C and Co_9_S_8_/Co‐Ni_3_S_2_||Pt/C electrolyzers. (c) Comparative analysis of Co_9_S_8_/Co‐Ni_3_S_2_||Pt/C and RuO_2_||Pt/C across different current densities and decay behaviors. (d) Electrolysis potentials at 100 mA cm^−2^ for a series of Ni‐based samples during total water splitting. (e) Stability tests of the RuO_2_||Pt/C and Co_9_S_8_/Co‐Ni_3_S_2_||Pt/C electrolyzers.

The practical potential of the low‐overpotential Co_9_S_8_/Co‐Ni_3_S_2_ OER catalyst was assessed by assembling an alkaline AEM electrolyzer to simulate an industrially relevant environment (Figure 7a and Figure S30). For commercial validation, an AEM electrolyzer was measured under identical conditions with a commercial RuO_2_ anode and a Pt/C cathode. A low cell voltage of 1.59 V was attained by the Co_9_S_8_/Co‐Ni_3_S_2_||Pt/C electrolyzer at 100 mA cm^−2^ under ambient conditions (Figure 7b). Compared with previously reported high‐efficiency Ni_3_S_2_‐derived catalysts intended for use at high current densities, the Co_9_S_8_/Co‐Ni_3_S_2_ catalyst exhibits even lower cell voltages at 100 mA cm^−2^ (Figure 7d and Table S4). During a 500 h continuous endurance test at 100 mA cm^−2^, the system demonstrated exceptional durability with a negligible voltage degradation rate of merely 0.16 mV h^−1^ (Figure 7e). Moreover, the hybrid catalyst demonstrated exceptional reproducibility with no discernible loss in its overall water splitting performance, accompanied by a Faradaic efficiency of approximately 100% (Figures S31 and S32). In comparison, the RuO_2_ electrolyzer showed poor durability at the same current density, with a sharp deterioration in stability within just 50 h of testing. Collectively, the excellent performance of Co_9_S_8_/Co‐Ni_3_S_2_ as the anode catalyst in AEM electrolyzers, as shown in Figure 7c, demonstrates its practical application potential for efficient and durable alkaline water splitting.

## 3 Conclusion

In conclusion, the Co_9_S_8_/Co‐Ni_3_S_2_ composite catalyst was synthesized via the one‐step method. It successfully verifies that the leaching and redeposition of Co ions exert a vital effect on boosting OER activity and stability. In contrast, leached Ni ions exert a negligible influence. Additionally, the catalyst surface becomes roughened after long‐term cycling, which strengthens mechanical robustness and enlarges the electrochemically active surface area simultaneously. Theoretical calculations further confirm that the Co_9_S_8_/Co‐Ni_3_S_2_ composite exhibits strong coupling between the Ni and Co 3d orbitals, facilitating faster electron transfer and enhanced catalytic activity, and that the catalyst favors the LOM. Notably, the catalyst demonstrates outstanding durability in an AEM electrolyzer, remaining stable for 500 h under 100 mA cm^−2^. The present study offers deeper insight into how metal ions dynamically leach and redeposit during the OER. It also offers a feasible methodology to fabricate electrocatalysts with both high activity and superior durability for water splitting in real applications, through the controlled harnessing of active site dynamics.

## Author Contributions

**Xue‐Min Chen**: conceptualization, supervision, project administration, writing – review and editing, writing – original draft, funding acquisition, methodology. **Xiu‐Li Zhao**: investigation, formal analysis, data curation. **Hui‐Ying Mu**: formal analysis, investigation, visualization. **Meng‐Ya Zhao**: investigation, formal analysis, data curation, writing – original draft. **Fa‐Tang Li**: writing – review and editing, funding acquisition, supervision. **Dong‐Ya Ma**: investigation, methodology, visualization. **Yue Zhou**: methodology, investigation.

## Funding

Central Government Guided Local Science and Technology Development Fund Project (264Z1107G), Natural Science Foundation of Hebei Province (B2021208005) and National Natural Science Foundation of China (51802075, 52301279 and 51901115).

## Conflicts of Interest

The authors declare no conflicts of interest.

## Supporting information




**Supporting File**: advs77217‐sup‐0001‐SuppMat.docx.

## Data Availability

The data that support the findings of this study are available from the corresponding author upon reasonable request.
