# Supplementary material for: Dynamic Cobalt Leaching‐Redeposition Equilibrium in Co9S8/Co‐Ni3S2 Electrocatalysts Boosts Oxygen Evolution Activity
Source: Adv Sci (Weinh). 2026 Aug 18;13(62):e77217. doi: 10.1002/advs.77217 (PMC13483130; doi:10.1002/advs.77217)
Supplement: Supplementary file 1 [file ADVS-13-e77217-s001.docx]

Supporting Information

**Dynamic Cobalt Leaching-Redeposition Equilibrium in Co_9_S_8_/Co-Ni_3_S_2_ Electrocatalysts Boosts Oxygen Evolution Activity**

Xue-Min Chen^1^*, Meng-Ya Zhao^1^, Xiu-Li Zhao^1^, Hui-Ying Mu^1^, Yue Zhou^2^, Dong-Ya Ma^1^, Fa-Tang Li^1^*

*Corresponding Authors

Email: chxm0058@163.com (Xue-Min Chen); lifatang@126.com (Fa-Tang Li)

^1^Hebei Provincial Key Laboratory of Photoelectric Control on Surface and Interface, College of Science, Hebei University of Science and Technology, Shijiazhuang, China

^2^College of Energy Storage Technology, Shandong University of Science and Technology, Qingdao, China

**Supplementary Texts**

**Reagents:**

Nickel foam was obtained from Suzhou Longshengbao Co., Ltd. Cobalt(II) nitrate hexahydrate (Co(NO_3_)_2_·6H_2_O), hydrochloric acid, Nickel(II) nitrate hexahydrate (Ni(NO_3_)_2_·6H_2_O), ethanol, Potassium hydroxide (KOH), Thioacetamide (C_2_H_5_NS) Tetramethylammonium Hydroxide (25% in Water), Ammonium fluoride (NH_4_F), and Urea (CO(NH_2_)_2_) were obtained from Innochem.

**Synthesis of Co_9_S_8_/Co-Ni_3_S_2，_ Ni_3_S_2_ and Co_9_S_8_：**

Firstly, Ni foam, which measures 20 × 30 mm in size and 1 mm in thickness, was treated in hydrochloric acid, ethanol and deionized water sequentially, with 10 minutes for each solvent. Taking Co_9_S_8_/Co-Ni_3_S_2_ as an example, 1.0 mmol of Co(NO_3_)_2_·6H_2_O, 2.0 mmol of C_2_H_5_NS were dissolved in 30 mL of deionized H_2_O, and the obtained solution was stirred magnetically for 0.5 h at room temperature to ensure homogeneity. Subsequently, the mixture, along with the pre-dried Ni foam, was sealed in a 50 mL autoclave and subjected to hydrothermal treatment at 150 °C for 15 h. Upon completion, the nickel foam substrate was retrieved, rinsed repeatedly using distilled water and ethanol in sequence, finally dried under vacuum to yield the Co_9_S_8_/Co-Ni_3_S_2_ catalyst. The preparation of Ni_3_S_2_ and Co_9_S_8_ was similar to that of Co_9_S_8_/Co-Ni_3_S_2_, except that the amount of Co(NO_3_)_2_·6H_2_O was varied to 0 and 2.3 mmol, separately.

**Synthesis of NiCo-LDH/Ni(OH)_2_ and NiO/NiCo_2_O_4_**:

0.6 mmol Ni(NO_3_)_2_·6H_2_O, 0.2 mmol Co(NO_3_)_2_·6H_2_O, 9 mmol CO(NH_2_)_2_ and 2.25 mmol NH_4_F were dissolved in 30 mL of deionized H_2_O, and the obtained solution was magnetically stirred for 0.5 h at room temperature to ensure homogeneity. Subsequently, the mixture, along with the pre-dried Ni foam, was sealed in a 50 mL autoclave and subjected to hydrothermal treatment at 90 °C for 6 h. Upon completion, the nickel foam substrate was retrieved, rinsed repeatedly using distilled water and ethanol in sequence, and finally dried under vacuum to yield the NiCoLDH/Ni(OH)_2_ catalyst. The as-prepared NiCoLDH/Ni(OH)_2_ catalyst was then transferred to a muffle furnace and subjected to annealing at 450 °C with a ramp rate of 5 °C min^-1^ for 3 h, affording the final product NiO/NiCo_2_O_4_.

**Characterization of Materials:**

The microstructure, phase constitution, and chemical structure were characterized using X-ray diffraction (XRD, Bruker D8), transmission electron microscopy (TEM, Talos F200X), Scanning Electron Microscope (SEM, SU8600), X-ray photoelectron spectra (XPS, Thermo ESCALAB 250XI), Inductively Coupled Plasma Optical Emission Spectrometer (ICP-MS, Agilent 5110), and Raman spectroscopy (Raman, KL325).

**Electrochemical analysis:**

All electrochemical characterizations were performed via a standard three-electrode conFigureuration connected to a CHI 660E analyzer; in this setup, Hg/HgO and a platinum sheet functioned as the reference and auxiliary electrodes, respectively. The OER activity was evaluated in a 1 M KOH solution. All potentials in this work are calibrated against the RHE scale using the relationship: *E*_RHE_ = *E*_Hg/HgO_ + 0.098 + 0.0591 × pH. The obtained LSV polarization curves were corrected for ohmic (iR) drop. The electrochemical impedance spectra (EIS) test applies to sinusoidal voltage using a 5 mV amplitude over a frequency range of 100 kHz to 0.01 Hz. The cyclic voltammetry (CV) tests were performed at scan rates of 20, 40, 60, 80, and 100 mV/s within the potential range of 0.08-0.18 V (vs. Hg/HgO). The double layer capacitance (C_dl_) was calculated by determining the slope of a linear regression of the anodic (i_a_) and cathodic (i_c_) current response to the scan rate for each sample.

**AEMWE tests:**

The anode of this AEM electrolyzer is an in-situ grown Co_9_S_8_/Co-Ni_3_S_2_ catalyst, the cathode is 0.2 mg cm^-2^ Pt/C ultrasonically sprayed onto Ni foam, and the anion exchange membrane is FAA-3-50. For comparison, commercial RuO_2_ powder coated on Ni foam is used as the anode. We use an AEM electrolysis water testing platform (YK-DJP-5L) to study the performance of the AEM electrolyzer in 1.0 M KOH electrolyte at room temperature and ambient pressure. The polarization curve is measured by linear sweep voltammetry (LSV) without real-time iR correction. The durability test is conducted by chronopotentiometry (CP) technology at a current density of 100 mA cm^-2^.

**Faradaic efficiency：**

In an AEM electrolyzer, we employed Co_9_S_8_/Co-Ni_3_S_2_ as the anode for water electrolysis and Pt/C as the cathode. The generated H₂ and O₂ gases were simultaneously collected at a current density of 100 mA·cm^-2^ using a simple water displacement method. The Faradaic efficiency was calculated according to the following formula:

$V_{theoretical}$ = $\frac{Q \times V_{m} \times1000}{ZF}$

η = $\frac{V_{measured}}{V_{theoretical}}$

**Theoretical calculations:**

All calculations were performed by using density functional theory (DFT) as implemented in the Vienna ab initio simulation package (VASP)^[1]^. The projector-augmented wave (PAW) potential was used to describe the electron-ion interactions^[2]^, and the Perdew-Burke-Ernzerhof (PBE) function of generalized gradient approximation (GGA) was used to describe the electron exchange and correlation energy^[3]^. The DFT-D3 empirical correction method was employed to describe van der Waals interactions^[4]^. The cutoff energy for plane-wave basis set was set as 500 eV. A vacuum layer larger than 15 was slected to eliminate interaction between two adjacent layers. All structures are fully relaxed until the maximal force on each atom is <0.03 eV/Å, and the electronic convergence criterion is 10^-5^ eV. The Gibbs free energy change were obtained based on the widely accepted OER four-electron transfer mechanism based on the previous report^[5]^.

In order to further reveal the effects on the OER activity, the D-band centers of Co_9_S_8_(440), Co-Ni_3_S_2_(110), and heterojunction based on Co-Ni_3_S_2_/Co_9_S_8_ is calculated.

The Gibbs free energy change for Co-NiOOH/CoOOH was calculated based on the AEM, following these basic steps:

* + OH^-^ → OH* + e^-^

OH* + OH^-^ → O* +H_2_O +e^-^

O* + OH^-^ → OOH* + e^-^

OOH* + OH^-^ → * + O_2_ + H_2_O +e**^-^**

The Gibbs free energy change for Co-NiOOH/CoOOH was calculated based on the LOM, following these basic steps:

OH* + OH^-^ → O* +H_2_O +e^-^

O* + OH^-^ → OOH* + e^-^

OOH* + OH^-^ → OO*+ H_2_O +e**^-^**

OO* → O_2_ +V_o_*

V_o_* + OH^-^ → OH*

The * represents the reaction site on the surface of the catalyst, and OH*, O* , V_o_*and OOH* denote the corresponding adsorbed intermediates. According to the method presented by Nørskov^[6]^, Gibbs free energy change is calculated using equation:

ΔG = ΔE + ΔZPE – TΔS

where ΔE is the electronic energy difference directly obtained from DFT calculations, ΔZPE is the change in zero-point energies, and TΔS obtained from the vibration analysis.

**Figures and curves:**

**Figure S1.** (a) SEM images of Co_9_S_8_/Co-Ni_3_S_2_. (b) Nanocoral regions corresponding to Co_9_S_8_; (c) Nanosheet regions corresponding to Co-Ni_3_S_2_.

(b)

(c)

**Figure S2.** The SEM-EDS spectrum and corresponding element ratios of Co_9_S_8_/Co-Ni_3_S_2_.

**Figure S3.** TEM image of Co_9_S_8_/Co-Ni_3_S_2_.

**Figure S4.** HRTEM images of Co_9_S_8_/Co-Ni_3_S_2_, corresponding enlarged images, and line intensity distributions of Ni_3_S_2_ and Co_9_S_8_.

**Figure S5.** HAADF-STEM image and corresponding element mappings of Co_9_S_8_/Co-Ni_3_S_2_.

**Figure S6.** SEM image of Co_9_S_8_.

**Figure S7.** SEM image of Ni_3_S_2_.

**Figure S8.** XRD patterns of Co_9_S_8_/Co-Ni_3_S_2_, Co_9_S_8_ and Ni_3_S_2_, including a magnification of the Co_9_S_8_/Co-Ni_3_S_2_ and Ni_3_S_2_ region.

**(c)**

**(b)**

**(a)**

**Figure S9.** XPS spectra of Co_9_S_8_/Co-Ni_3_S_2_ and Ni_3_S_2_.

**(b)**

**(a)**

**Figure S10.** (a) LSV curves; (b) Corresponding Tafel slopes of Co_9_S_8_/Co-Ni_3_S_2_ and contrast samples.

**Figure S11.** Comprehensive comparison of OER overpotentials at different current densities and Tafel slopes.

**Figure S12.** The LSV curves of Ni_3_S_2_ before and after test.

**Figure S13.** The LSV curves of Co_9_S_8_ before and after test.

**Figure S14.** SEM images corresponding to different OER reaction durations.

|  |  |
| --- | --- |
| **0 h** | **1 h** |
|  |  |
| **1.5 h** | **2.5 h** |
|  |  |
| **4 h** | **6 h** |

**Figure S15.** Elemental mapping images of the catalysts before and after different OER intervals.

**(b)**

**(a)**

**Figure S16.** (a) CV curves of Ni_3_S_2_ before and after 150 cycles in 1 M KOH；(b) CV curves of Co_9_S_8_ before and after 150 cycles in 1 M KOH.

**Figure S17.** XRD patterns and chronopotentiometry curves at 50 mA cm^-2^ (without prior CV activation) of NiO/NiCo_2_O_4_ (a-b) and NiCo-LDH/Ni(OH)_2_ (c-d).

**(b)**

**(a)**

**Figure S18.** CV curves of Ni₃S₂ at different scan rates and corresponding C_dl_.

**(b)**

**(a)**

**Figure S19.** CV curves of Co_9_S_8_ at different scan rates and corresponding C_dl_.

**(b)**

**(a)**

**Figure S20.** CV curves of Co_9_S_8_/Co-Ni_3_S_2_ at different scan rates and corresponding C_dl_.

**(b)**

**(a)**

**Figure S21.** EIS Nyquist plots of the catalysts.

**Figure S22.** Bode phase plots of the in situ electrochemical impedance spectra of Co_9_S_8_ for OER.

**Figure S23.** In-situ Raman spectra of Ni_3_S_2_ at different potentials.

**Figure S24.** Comparison of LSV curves of Ni_3_S_2_ in 1 M KOH and 1 M TMAOH.

**Figure S25.** Comparison of LSV curves of Co_9_S_8_ in 1 M KOH and 1 M TMAOH.

**Figure S26.** The computational models of Co-Ni_3_S_2_、Co_9_S_8_ and Co_9_S_8_/Co-Ni_3_S_2_. The gray spheres represent Ni atoms, the yellow spheres represent S atoms, and the blue spheres represent Co atoms.

**Figure S27.** XPS spectra of Co_9_S_8_/Co-Ni_3_S_2_ before and after stability test.

**Figure S28.** The computational models of AEM mechanism.

**Figure S29.** The computational models of LOM mechanism. The gray spheres represent Ni atoms, the pink spheres represent H atoms, the red spheres represent O atoms and the blue spheres represent Co atoms.

**Figure S30.** Assembly diagram of the AEM electrolyzer for water electrolysis.

**Figure S31.** The Faradaic efficiency of Co_9_S_8_/Co-Ni_3_S_2_||Pt/C electrolyzers.

**Figure S32.** The reproducibility of Co_9_S_8_/Co-Ni_3_S_2_||Pt/C electrolyzers.

**Table S1** XPS analysis of the Co_9_S_8_/Co-Ni_3_S_2_ catalyst after various OER reaction durations.

| **OER reaction time ( h)** | **Ni^2+^/Ni^3+^** | **Co^2+^/Co^3+^** |
| --- | --- | --- |
| 0.5 | 2.97 | 0.78 |
| 1 | 2.23 | 0.48 |
| 1.5 | 2.03 | 0.46 |
| 2.5 | 2.0 | 0.39 |
| 4 | 1.39 | 0.37 |
| 6 | 1.17 | 0.35 |

**Table S2** The comparison of OER performances of Co_9_S_8_/Co-Ni_3_S_2_ and other recently-reported Ni-based catalysts.

| **Catalysts** | **Overpotential**  **(mV)** | **Ref** |
| --- | --- | --- |
| **Co_9_S_8_/Co-Ni_3_S_2_** | **280 at 50 mA cm^-2^** | **This work** |
| **Co_9_S_8_/Co-Ni_3_S_2_** | **300 at 100 mA cm^-2^** | **This work** |
| Zn–NiS-3 | 320 at 50 mA cm^-2^ | [7] |
| Mo-NiS_x_/NF | 307 at 50 mA cm^-2^ | [8] |
| Ni_3_S_2_@Ni_5_P_4_ | 399 at 50 mA cm^-2^ | [9] |
| Fe-NiS/NiS_2_ | 361 at 100 mA cm^-2^ | [10] |
| Fe-Ni_3_S_2_/Ni_2_P | 357 at 100 mA cm^-2^ | [11] |
| Co-Ni_3_S_2_/NF | 459 at 50 mA cm^-2^ | [12] |
| Ni_3_S_2_-Co_9_S_8_/NCA | 337 at 10 mA cm^-2^ | [13] |
| CuCo-Ni_3_S_2_/NF | 400 at 100mA cm^-2^ | [14] |
| VO_x_/NiS/NF | 330 at 50 mA cm^-2^ | [15] |
| Co_9_S_8_@CoS_2_ | 396 at 50 mA cm^-2^ | [16] |
| Co_9_S_8_-CoSe_2_ | 340 at 10 mA cm^-2^ | [17] |

**Table S3** Stability comparison under three-electrode conditions.

| **Catalysts** | **j (mA/cm^2^)** | **Time (h)** | **Ref.** |
| --- | --- | --- | --- |
| Co_9_S_8_/Co-Ni_3_S_2_ | 100 | 250 | This work |
| Ni_3_S_2_-W-V_S_ | 100 | 48 | [18] |
| Fe-Ni_3_S_2_/MoO_x_ | 100 | 24 | [19] |
| Co_9_S_8_@FeS_2_ | 20 | 24 | [20] |
| Fe–Ni_3_S_4_/Cr_2_O_3_ | 10 | 100 | [21] |
| Ce_0.2_-Ni_3_S_4_ | 20 | 24 | [22] |
| Co_9_S_8_/Ni_3_S_2_@NF | 20 | 20 | [23] |
| Fe,Ce-Ni_3_S_2_@NF | 20 | 50 | [24] |
| FcDA-Ni_3_S_2_/NF | 10 | 24 | [25] |
| NiS/FeS_2_ | 10 | 80 | [26] |
| Co_9_S_8_-Ag-Ni_3_S_2_@NF | 10 | 18 | [27] |
| Ni_3_S_2_–FeMoO_4_/NFF | 100/500 | 40/50 | [28] |
| Mn−Ni_3_S_2_@MoS_2_ | 100 | 50 | [29] |
| Fe-NiO/NiS_2_ | 10 | 100 | [30] |

**Table S4** Comparison of the cell potential of this report with several bifunctional Ni_3_S_2_-based electrocatalysts.

| **Catalysts** | **Cell voltage (V)**  **@100 mA/cm^2^** | **Cell voltage (V)**  **@500 mA/cm^2^** | **Ref.** |
| --- | --- | --- | --- |
| **Co_9_S_8_/Co-Ni_3_S_2_** | **1.59** | **1.80** | **This work** |
| Fe-Ni_3_S_2_/MoO_x_ | 1.75 | ---- | [19] |
| NiFeP/Ni_3_S_2_ | ---- | 2.24 | [31] |
| PBSCF-Ni_3_S_2_ | 1.61 | 1.76 | [32] |
| Mn−Ni_3_S_2_@MoS_2_ | 1.81 | ---- | [29] |
| ZnCoNiS | 1.85 | ---- | [33] |
| MoS_2_-Ni_3_S_2_/NF | 2.4 | ---- | [34] |
| Fe/W-Ni_3_S_2_ | 1.80 | 2.05 | [35] |
| CoMoP/Ni_3_S_2_ | 1.87 | ----- | [36] |
| NiFeLDH/Fe_2_O_3_/Ni_3_S_2_/IF | 1.80 | ----- | [37] |
| Vs-Ni_3_S_2_/Fe_2_P | 1.76 | ----- | [38] |

**Supplementary References**

1. G. Kresse, J. Furthmiiller, Efficiency of ab-initio total energy calculations for metals and semiconductors using a plane-wave basis set Comput. Mater. Sci., 6 (1996) 15-50.
2. G. Kresse, D. Joubert, From ultrasoft pseudopotentials to the projector augmented-wave method, Phys. Rev. B, 59 (1998) 1758-1775.
3. J.P. Perdew, K. Burke, M. Ernzerhof, Generalized Gradient Approximation Made Simple, Phys. Rev. Lett., 77 (1996) 3865-3868.
4. Stefan Grimme, Jens Antony, S. Ehrlich, H. Krieg, A consistent and accurate ab initio parametrization of density functional dispersion correction (DFT-D) for the 94 elements H-Pu, J. Chem. Phys., 132 (2010) 154104.
5. HanhuiYou, DongshuangWu, DuanhuiSi, MinnaCao, FanfeiSun, HaoZhang, HuiMinWang, Tian-FuLiu, RongCao, Monolayer NiIr-Layered Double Hydroxide as a Long-Lived Efficient Oxygen Evolution Catalyst for Seawater Splitting, J. Am. Chem. Soc., 144 (2022) 9254-9263.
6. J.K. Nùrskov, J. Rossmeisl, A. Logadottir, L. Lindqvist, Origin of the Overpotential for Oxygen Reduction at a Fuel-Cell Cathode, J. Phys. Chem. B, 108 (2004) 17886-17892.
7. C. Prakash, P. Sahoo, R. Yadav, A. Pandey, V.K. Singh, A. Dixit, Nanoengineered Zn-modified Nickel Sulfide (NiS) as a bifunctional electrocatalyst for overall water splitting, Int. J. Hydrogen Energy, 48 (2023) 21969-21980.
8. Y. Zhang, M. Chen, P. Guo, Y. Du, B. Song, X. Wang, Z. Jiang, P. Xu, Magnetic field‐enhanced water splitting enabled by bifunctional molybdenum‐dopednickel sulfide on nickel foam, Carbon Energy, 5 (2023) e351.
9. S. Khan, T. Ali, X. Wang, W. Iqbal, T. Bashir, W. Chao, H. Sun, H. Lu, C. Yan, R. Muhammad Irfan, Ni_3_S_2_@Ni_5_P_4_ nanosheets as highly productive catalyst for electrocatalytic oxygen evolution, Chem. Eng. Sci., 247 (2022) 117020.
10. X. Zhao, X. Wang, L. Chen, X. Kong, Z. Li, Y. Zhao, Z. Wu, T. Wang, Z. Liu, P. Yang, The design and synthesis of Fe doped flower-like NiS/NiS_2_ catalyst with enhanced oxygen evolution reaction, J. Electroanal. Chem., 920 (2022) 116630.
11. X. Wang, X. Yu, J. Bai, G. Yuan, P. He, Y. Zhu, S. Wu, F. Qin, L. Ren, Interface engineering assisted Fe-Ni_3_S_2_/Ni_2_P heterostructure as a high-performance bifunctional electrocatalyst for OER and HER, Electrochim. Acta, 458 (2023) 142524.
12. X. Wang, S. Wang, S. Chen, P. He, Y. Xu, L. Jia, D. Yang, X. He, H. Deng, B. Jia, H. Zhang, H. Liu, Facile one-pot synthesis of binder-free nano/micro structured dendritic cobalt activated nickel sulfide: a highly efficient electrocatalyst for oxygen evolution reaction, Int. J. Hydrogen Energy, 45 (2020) 19304-19312.
13. X. Wang, Y. Yang, R. Wang, L. Li, X. Zhao, W. Zhang, Porous Ni_3_S_2_-Co_9_S_8_ Carbon Aerogels Derived from Carrageenan/NiCo-MOF Hydrogels as an Efficient Electrocatalyst for Oxygen Evolution in Rechargeable Zn-Air Batteries, Langmuir, 38 (2022) 7280-7289.
14. J.-F. Qin, M. Yang, S. Hou, B. Dong, T.-S. Chen, X. Ma, J.-Y. Xie, Y.-N. Zhou, J. Nan, Y.-M. Chai, Copper and cobalt co-doped Ni_3_S_2_ grown on nickel foam for highly efficient oxygen evolution reaction, Appl. Surf. Sci., 502 (2020) 144172.
15. Y.-M. Chai, X.-Y. Zhang, J.-H. Lin, J.-F. Qin, Z.-Z. Liu, J.-Y. Xie, B.-Y. Guo, Z. Yang, B. Dong, Three-dimensional VO_x_/NiS/NF nanosheets as efficient electrocatalyst for oxygen evolution reaction, Int. J. Hydrogen Energy, 44 (2019) 10156-10162.
16. Y. Dong, J. Ran, Q. Liu, G. Zhang, X. Jiang, D. Gao, Hydrogen-etched CoS_2_ to produce a Co_9_S_8_@CoS_2_ heterostructure electrocatalyst for highly efficient oxygen evolution reaction, RSC Adv, 11 (2021) 30448-30454.
17. S. Chakrabartty, S. Karmakar, C.R. Raj, An Electrocatalytically Active Nanoflake-Like Co_9_S_8_-CoSe_2_ Heterostructure for Overall Water Splitting, ACS Appl. Nano Mater., 3 (2020) 11326-11334.
18. Z. Wang, S. Shen, Z. Lin, W. Tao, Q. Zhang, F. Meng, L. Gu, W. Zhong, Regulating the Local Spin State and Band Structure in Ni_3_S_2_ Nanosheet for Improved Oxygen Evolution Activity, Adv. Funct. Mater., 32 (2022) 2112832.
19. N.S. Gultom, C.-H. Li, D.-H. Kuo, M.Z. Silitonga, Multiphase Fe-doped Ni_3_S_2_/MoO_x_ electrocatalyst prepared by facile one-step hydrothermal for full-cell water splitting: Effect of Mo on physical and electrochemical properties, Appl. Catal. B, 353 (2024) 124100.
20. W. Liu, Z. Teng, B. An, J. Dong, N. Li, Y. Gao, L. Ge, Asymmetric electronic structure effects in Co_9_S_8_@FeS_2_ core-shell nanostructures for highly efficient overall water splitting by modulating d-p orbital hybridization, Chem. Eng. J., 504 (2025) 158932.
21. K. Wang, C. Ni, L. Jin, X. Qian, H. Xu, H. Chen, G. He, Fe doping intensifies the built-in electric field for tailoring the reconstruction of sulfides towards efficient oxygen evolution, Chem. Sci., 16 (2025) 7467-7476.
22. C. Gao, L. Pan, H. Wang, H. Guo, S. Melhi, M.A. Amin, J. Lin, Cerium Doping-Induced Enrichment of Ni_3_S_4_ Phase for Boosting Oxygen Evolution Reaction, ChemSusChem, 17 (2024) e202400056.
23. C. Gao, L. Kong, L. Pan, D. Li, J. Lin, A novel sacrificial solvent method to synthesize self-supporting Co_9_S_8_/Ni_3_S_2_ heterostructure catalyst for efficient oxygen evolution reaction, J. Colloid Interface Sci., 652 (2023) 1756-1763.
24. Y. He, K. Shi, X. Wang, X. Zheng, L. Luo, L. Lin, Z. Sun, G. Sun, Synergistic Fe,Ce doping of Ni_3_S_2_ for enhancing oxygen evolution reaction performance, Inorg. Chem. Front., 11 (2024) 7623-7632.
25. L. Jin, C. Yang, S. Chen, J. Hou, P. Liu, Ligand-Induced Electronic Structure Modulation of Self-Evolved Ni_3_S_2_ Nanosheets for the Electrocatalytic Oxygen Evolution Reaction, Inorg. Chem., 63 (2024) 9967-9974.
26. X. Yu, J. Mei, Y. Du, X. Cheng, X. Wang, Q. Wu, Engineered interface of three-dimensional coralliform NiS/FeS_2_ heterostructures for robust electrocatalytic water cleavage, Nano Res., 17 (2023) 4693-4701.
27. T. Tadesse Tsega, Y. Zhang, J. Zai, C.W. Lai, X. Qian, Incorporation of Ag in Co_9_S_8_-Ni_3_S_2_ for Predominantly Enhanced Electrocatalytic Activities for Oxygen Evolution Reaction: A Combined Experimental and DFT Study, Chempluschem, 89 (2024) e202400235.
28. H. Chen, J. Zhang, R. Wan, X. Zhang, Q. Pan, M. Li, B. Chen, One-step hydrothermal synthesis of a Ni_3_S_2_-FeMoO_4_ nanowire-nanosheet heterostructure array for synergistically boosted oxygen evolution reaction, RSC Adv., 14 (2024) 9109-9113.
29. S. Ye, Z. Du, Z. Cai, D. Ou, H. Guo, Q. Liu, W. Yang, Q. Shi, Mn-Doped Crystalline Ni_3_S_2_/Amorphous MoS_2_ Core–Shell Nanorods as Bifunctional Electrocatalysts for Highly-Efficient Overall Water Splitting, ACS Appl. Nano Mater., 7 (2024) 3096-3104.
30. N. Zhang, Y. Hu, L. An, Q. Li, J. Yin, J. Li, R. Yang, M. Lu, S. Zhang, P. Xi, C.H. Yan, Surface Activation and Ni-S Stabilization in NiO/NiS_2_ for Efficient Oxygen Evolution Reaction, Angew. Chem. Int. Ed. Engl., 61 (2022) e202207217.
31. Y. Song, X. Zhang, Z. Xiao, Y. Wang, P. Yi, M. Huang, L. Zhang, Coupled amorphous NiFeP/cystalline Ni_3_S_2_ nanosheets enables accelerated reaction kinetics for high current density seawater electrolysis, Appl. Catal. B, 352 (2024) 124028.
32. S. Jeong, U. Kim, S. Lee, Y. Zhang, E. Son, K.-J. Choi, Y.-K. Han, J.M. Baik, H. Park, Superaerophobic/Superhydrophilic Multidimensional Electrode System for High-Current-Density Water Electrolysis, ACS Nano, 18 (2024) 7558-7569.
33. P. Li, L. Zhang, Y. Yao, T. Xie, W. Du, T. Zhao, J. Jiang, ZnCoNiS nanoflowers electrodes with rich heterointerface as efficient bifunctional electrocatalyst for overall water splitting, Int. J. Hydrogen Energy, 51 (2024) 1521-1533.
34. Y. Kumar, S. Barik, N.S. Samudre, G.P. Kharabe, I. Chauhan, N. Manna, S. Bhat, S. Kurungot, Self‐Supported Super‐Hydrophilic Interconnected Nanospikes and Particles of MoS_2_‐Ni_3_S_2_/NF with Optimum d‐Band Center for Anion Exchange Membrane Water Electrolyzer, Adv. Sustain. Syst., 9 (2025) 2400957.
35. S. Wang, D. Yuan, S. Sun, S. Huang, Y. Wu, L. Zhang, S.X. Dou, H.K. Liu, Y. Dou, J. Xu, Iron, Tungsten Dual‐Doped Nickel Sulfide as Efficient Bifunctional Catalyst for Overall Water Splitting, Small, 20 (2024) 202311770.
36. M.B. Poudel, N. Logeshwaran, A.R. Kim, K. S.C, S. Vijayapradeep, D.J. Yoo, Integrated core-shell assembly of Ni_3_S_2_ nanowires and CoMoP nanosheets as highly efficient bifunctional electrocatalysts for overall water splitting, J. Alloys Compd., 960 (2023) 170678.
37. D. Deng, Q. Li, S. Lei, W. Zhang, H. Li, L. Xu, NiFe LDH/Fe_2_O_3_/Ni_3_S_2_ Heterostructure with a Superhydrophilic/Superaerophobic Surface for Solar-Driven Electrolytic Water Splitting, Inorg. Chem., 63 (2024) 20022-20029.
38. N. Long, J. Peng, Y. Jiang, W. Shen, R. He, M. Li, Synergy of Interface Coupling and Sulfur Vacancies in Ni_3_S_2_/Fe_2_P for Water Splitting, Inorg. Chem., 63 (2024) 16382-16392.
